# Supplementary material for: How much do the physician review and InterVA model agree in determining causes of death? a comparative analysis of deaths in rural Ethiopia
Source: BMC Public Health. 2015 Jul 15;15:669. doi: 10.1186/s12889-015-2032-7 (PMC4503295; doi:10.1186/s12889-015-2032-7)
Supplement: Additional file 1: — Categorization of causes of death from the physicians review and InterVA model, KA-HDSS, 2014. [file 12889_2015_2032_MOESM1_ESM.docx]

**Supplementary file 1**: Categorization of causes of death from the physicians review and InterVA model, KA-HDSS, 2014

| **InterVA COD list** | **COD-common to both methods (specific)** | **COD-common to both methods (broad category)** |
| --- | --- | --- |
| Sepsis (non-obstetric) | Pneumonia/sepsis | Communicable diseases |
| Acute resp infect incl pneumonia | Acute lower respiratory Tract infections incl pneumonia | Communicable diseases |
| HIV/AIDS related death | HIV/AIDS | Communicable diseases |
| Diarrhoeal diseases | Infectious/diarrhoeal/parasitic | Communicable diseases |
| Malaria | Malaria | Communicable diseases |
| Meningitis and encephalitis | Meningitis | Communicable diseases |
| Pulmonary tuberculosis | TB | Communicable diseases |
| Other and unspecified infect dis | Infectious/diarrhoeal/parasitic | Communicable diseases |
| Digestive neoplasms | Neoplasm | Non Communicable diseases |
| Respiratory neoplasms | Neoplasm | Non Communicable diseases |
| Reproductive neoplasms MF | Neoplasm | Non Communicable diseases |
| Other and unspecified neoplasms | Neoplasm | Non Communicable diseases |
| Severe anaemia | Malnutrition | Non Communicable diseases |
| Severe malnutrition | Malnutrition | Non Communicable diseases |
| Diabetes mellitus | Other non-communicable diseases | Non Communicable diseases |
| Acute cardiac disease | Cardiovascular | Non Communicable diseases |
| Stroke | Cardiovascular | Non Communicable diseases |
| Other and unspecified cardiac dis | Cardiovascular | Non Communicable diseases |
| Chronic obstructive pulmonary disorder | Chronic obstructive pulmonary disorder | Non Communicable diseases |
| Asthma | Chronic obstructive pulmonary disorder | Non Communicable diseases |
| Acute abdomen | Gastrointestinal disorders | Non Communicable diseases |
| Liver cirrhosis | Gastrointestinal disorders | Non Communicable diseases |
| Renal failure | Renal disorder | Non Communicable diseases |
| Epilepsy | Mental | Non Communicable diseases |
| Obstetric haemorrhage | Maternal | Maternal/Neonatal |
| Prematurity | Perinatal | Maternal/Neonatal |
| Birth asphyxia | Perinatal | Maternal/Neonatal |
| Neonatal pneumonia | Perinatal | Maternal/Neonatal |
| Neonatal sepsis | Perinatal | Maternal/Neonatal |
| Other and unspecified neonatal Co | Perinatal | Maternal/Neonatal |
| Road traffic accident | Accidents/injuries | Accidents/injuries |
| Other transport accident | Accidents/injuries | Accidents/injuries |
| Accidental fall | Accidents/injuries | Accidents/injuries |
| Accidental drowning and submersion | Accidents/injuries | Accidents/injuries |
| Intentional self-harm | Accidents/injuries | Accidents/injuries |
| Assault | Accidents/injuries | Accidents/injuries |
| Exposure to force of nature | Accidents/injuries | Accidents/injuries |
| Other and unspecified external Co | Accidents/injuries | Accidents/injuries |
| Other and unspecified NCD | Other non-communicable diseases | Non Communicable diseases |
| Indeterminate | Undetermined | Undetermined |

| **Physician review COD list** | **Common COD (Specific )** | **COD-common to both methods (Broad Category)** |
| --- | --- | --- |
| Accident unspecified | Accidents/injuries | Accidents/injuries |
| Accidental drowning and submersion | Accidents/injuries | Accidents/injuries |
| Accidental Fall | Accidents/injuries | Accidents/injuries |
| Accidental Exposure to Smoke, Fire and Flames | Accidents/injuries | Accidents/injuries |
| Assault | Accidents/injuries | Accidents/injuries |
| Contact with Venomous animals and Plants | Accidents/injuries | Accidents/injuries |
| Exposure to force of nature | Accidents/injuries | Accidents/injuries |
| Intentional Self Harm | Accidents/injuries | Accidents/injuries |
| other transport accident | Accidents/injuries | Accidents/injuries |
| Pedestrian injured in Traffic Accident | Accidents/injuries | Accidents/injuries |
| War Death | Accidents/injuries | Accidents/injuries |
| Acute lower respiratory infections (including pneumonia and acute bronchitis) | Acute lower respiratory Tract infections incl pneumonia | Communicable diseases |
| Asthma | Chronic obstructive pulmonary disorder | Non Communicable diseases |
| Chronic obstructive lung Disease | Chronic obstructive pulmonary disorder | Non Communicable diseases |
| Cerebrovascular disease | Cardiovascular | Non Communicable diseases |
| Congestive Heart Failure | Cardiovascular | Non Communicable diseases |
| Hypertensive Diseases | Cardiovascular | Non Communicable diseases |
| Ischemic heart disease | Cardiovascular | Non Communicable diseases |
| other specified Disorders of the nervous system | Cardiovascular | Non Communicable diseases |
| Acute abdomen | Gastrointestinal disorders | Non Communicable diseases |
| Chronic liver disease | Gastrointestinal disorders | Non Communicable diseases |
| Disorder of kidney & ureter | Gastrointestinal disorders | Non Communicable diseases |
| Gastric and duodenal ulcer | Gastrointestinal disorders | Non Communicable diseases |
| Paralytic ileus and intestinal obstruction | Gastrointestinal disorders | Non Communicable diseases |
| HIV/AIDS | HIV/AIDS | Communicable diseases |
| Viral hepatitis | Infectious/diarrhoea/parasitic | Communicable diseases |
| Intestinal infection disease (including diarrhoeal diseases) | Infectious/diarrhoea/parasitic | Communicable diseases |
| Typhoid and paratyphoid | infectious/diarrhoea/parasitic | Communicable diseases |
| Malaria | Malaria | Communicable diseases |
| Nutritional anaemia | Malnutrition | Non Communicable diseases |
| other specified endocrine disorder | Malnutrition | Non Communicable diseases |
| Severe malnutrition | Malnutrition | Non Communicable diseases |
| other direct maternal causes unspecified | Maternal | Maternal/Neonatal |
| post-partum haemorrhage | Maternal | Maternal/Neonatal |
| Measles | Measles | Communicable diseases |
| Meningitis | Meningitis | Communicable diseases |
| Alzheimer disease | Mental | Non Communicable diseases |
| Epilepsy | Mental | Non Communicable diseases |
| Mental disorder unspecified | Mental | Non Communicable diseases |
| malignant neoplasm of oesophagus | Neoplasm | Non Communicable diseases |
| Malignant neoplasm of stomach | Neoplasm | Non Communicable diseases |
| Malignant neoplasm of breast | Neoplasm | Non Communicable diseases |
| Malignant neoplasm of oesophagus | Neoplasm | Non Communicable diseases |
| Malignant neoplasm of Lymphoid, hematopoietic & related tissue | Neoplasm | Non Communicable diseases |
| Malignant neoplasm of lymphoid Hematopoietic & related tissue | Neoplasm | Non Communicable diseases |
| Malignant neoplasm of prostate | Neoplasm | Non Communicable diseases |
| Malignant neoplasm of small & large intestine | Neoplasm | Non Communicable diseases |
| Malignant neoplasm of Stomach | Neoplasm | Non Communicable diseases |
| Malignant neoplasm of trachea, bronchus and lung | Neoplasm | Non Communicable diseases |
| neoplasm of uncertain or unknown behaviour ,unspecified | Neoplasm | Non Communicable diseases |
| other specified neoplasms | Neoplasm | Non Communicable diseases |
| Diabetes mellitus | ONCD | Non Communicable diseases |
| birth asphyxia respiratory birth disorder | Perinatal | Maternal/Neonatal |
| Birth asphyxia and perinatal respiratory disorders | Perinatal | Maternal/Neonatal |
| Congenital malformation and other unspecified | Perinatal | Maternal/Neonatal |
| Congenital malformation of nervous system | Perinatal | Maternal/Neonatal |
| other disease related to the perinatal period Unspecified | Perinatal | Maternal/Neonatal |
| Prematurity(including respiratory distress) | Perinatal | Maternal/Neonatal |
| Still birth | Perinatal | Maternal/Neonatal |
| Bacterial sepsis of new born | Perinatal | Maternal/Neonatal |
| Neonatal Pneumonia | Perinatal | Maternal/Neonatal |
| Renal failure | Renal | Non Communicable diseases |
| Tuberculosis | TB | Communicable diseases |
| Undetermined | Undetermined | Undetermined |
